# Immune checkpoint inhibitor-induced myocarditis and myositis in liver cancer patients: A case report and literature review

**DOI:** 10.3389/fonc.2022.1088659

**Published:** 2023-01-11

**Authors:** Haoran Mei, Wu Wen, Kang Fang, Yuanpeng Xiong, Weiqi Liu, Jie Wang, Renhua Wan

**Affiliations:** ^1^Department of General Surgery, The First Affiliated Hospital of Nanchang University, Nanchang, China; ^2^Department of General Surgery, The First Affiliated Hospital of Gannan Medical University, Ganzhou, China

**Keywords:** hepatocellular carcinoma, immune checkpoint inhibitors, irAEs (immune-related adverse events), immune myositis, immune myocarditis

## Abstract

With the development of immunotherapy, immune checkpoint inhibitors (ICIs) are widely used in clinical oncology and have achieved good results. ICIs could induce immune-related adverse events (irAEs) in cancer treatment, which warrant sufficient attention. Among them, immune myositis can manifest severe symptoms affecting the whole body, and immune myocarditis occurs with a low incidence but high fatality rate. Here we report a case of grade 3/4 adverse reactions in a patient with partial hepatectomy for malignancy after using ICIs and describe the clinical presentation, laboratory results, treatment, and prognosis. It emphasizes that clinicians should focus on being alert to irAEs in liver cancer patients who have received ICI therapy. The case we present is a 56-year-old male diagnosed with hepatocellular carcinoma. Right hepatic lobectomy was performed in April 2019. Postoperative follow-up showed that transcatheter arterial chemoembolization (TACE) combined with sorafenib (400 mg twice daily) failed to stop the recurrence of the tumor. In December 2020, the patient started to use Camrelizumab injections (200mg/injection every 21 days as a cycle). After 3 cycles, the patient had decreased muscle strength in both lower extremities with chest tightness, dyspnea, and expectoration (whitish sputum). The diagnosis was ICIs injection-induced immune myocarditis and myositis accompanied. The patient’s condition improved considerably by steroid pulse therapy timely. The case emphasizes that clinicians should focus on being alert to irAEs in liver cancer patients who have received ICI therapy.

## Introduction

Primary liver cancer is among the most frequent solid tumor types that seriously threaten human health and is a leading cause of cancer-related death worldwide. According to GLOBOCAN 2020, there were approximately 910000 new cancer cases and around 830000 deaths in 2020 (1). Hepatocellular carcinoma (HCC) is the most common type of primary liver cancer. Traditional treatment modalities include surgical resection, TACE, radiofrequency ablation (RFA), targeted therapy, etc. Although these methods can effectively address local lesions, they cannot eliminate all cancer cells. The residual cancer cells can lead to the recurrence and metastasis of malignant liver tumors. Recently, immunotherapy has been widely used to treat hepatocellular carcinoma (HCC). Many clinical practices or clinical studies are based on ICIs, including local treatment combined with immunotherapy, targeted therapy combined with immunotherapy, adjuvant immunotherapy following surgical resection, combination therapy of a double immune checkpoint, etc. Multiple studies have demonstrated that combining different treatment modalities with immunotherapies may represent an effective therapeutic strategy for HCC (2–5). The objective response rate (ORR) of combination therapy based on ICIs can reach approximately 30% (3, 4).

ICIs also known as immune checkpoint blockade (ICB). The primary reliable targets of HCC immunotherapy include programmed death receptor 1 (PD-1)/programmed death receptor ligand1 (PD-L1) and cytotoxic T-lymphocyte antigen 4 (CTLA-4) (6). In HCC immunotherapy, the anti-PD-1 monoclonal antibodies include Nivolumab, Pembrolizumab, Camrelizumab, and Tislelizumab; the anti-PD-L1 monoclonal antibodies include Atezolizumab, Durvalumab, and Sintilimab; the anti-CTLA-4 monoclonal antibodies include Ipilimumab and Tremelimumab. ICIs do not kill tumors directly but can activate the host’s immune system to generate effects in the immune microenvironment (TME), thereby decreasing the risk of local recurrence and distant metastasis (7). ICIs remove the standard inhibitory control that negatively regulates T-cell function in HCC treatment, but they may also induce T-cell hyper-activation and immune-related adverse events (irAEs) (8). These irAEs can affect all organs, including the skin, lungs, thyroid, digestive system, nervous system, musculoskeletal system, etc. Immune myocarditis and myositis are uncommon occurrences with potentially serious outcomes.

In 2016, Johnson et al. was first to report two cases of fulminant myocarditis following treatment with ICIs (9). They systematically described the incidence of myocarditis in a retrospective clinical trial population. The largest single-center series of immune myositis patients who received ICIs were also reported by Jeffrey Aldrich et al. in 2021 (10). Both immune myocarditis and immune myositis are uncommon occurrences with potentially serious outcomes. Compared with other tumors, the incidence of irAEs was not significantly different in HCC patients but tended to increase (11). Liver cancer, immune myocarditis and immune myositis co-occur in the same individual is rare. This case was reported immune myocarditis and myositis in a patient with partial hepatectomy for malignancy after using ICIs. We present the following case in accordance with the CARE reporting checklist.

## Case description

**Figure 1:**
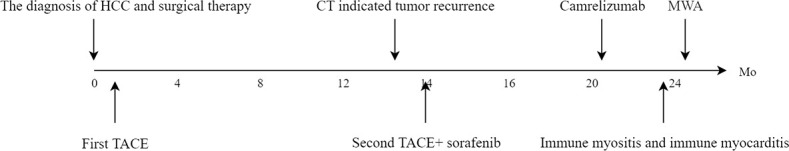
Treatment course.

**Figure 2:**
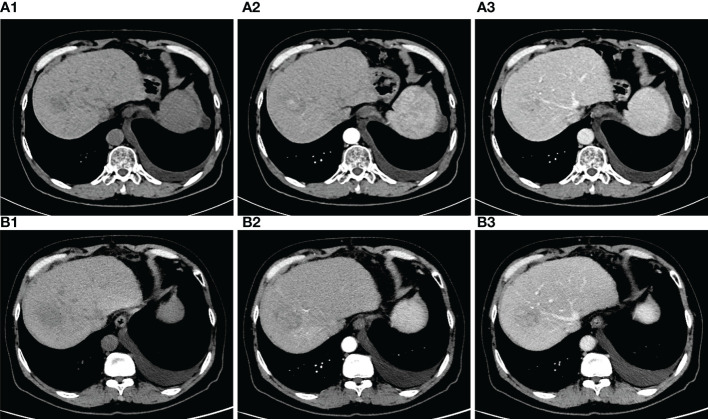
**(A) (A1)** plain phase **(A2)** arteria phase **(A3)** venous phase. Red arrows: tumor nodules. CT of the abdomen scan revealed that, nodules were observed with uneven density. On the enhanced scan, the nodules showed obvious enhancement, and no the venous and delayed scan showed slightly lower density. **(B) (B1)** plain phase **(B2)** arteria phase **(B3)** venous phase. Compared with the last CT, the nodules were bigger than before.

A 56-year-old male was admitted to the hospital because of dyspnea and chest tightness for 20 days (**Figure 1**). He had no previous history of autoimmune diseases or steroid medication. His personal history, and family history were negative. The vital signs of the patient were normal on admission. The patient with HCC in the right lobe of the liver was treated with a partial hepatectomy on April 2, 2019. The postoperative pathological results in poorly differentiated hepatocellular carcinoma and negative margins. On May 14, 2019, the patient underwent TACE because his microvascular infiltration (MVI) grade is M2. On May 26, 2020, abdominal contrast-enhanced computed tomography (CT) reexamination revealed multiple nodular enhancements, which could suggest tumor recurrence (**Figure 2A**). The patient received the second TACE combined with sorafenib (400 mg twice daily) on June 9, 2020. After six months, the enhanced CT scan revealed that the more enhanced nodules were bigger than before (**Figure 2B**). Consequently, he received treatment with the TACE again and started to use Camrelizumab injections (200mg/injection every 21 days as a cycle) on Dec 29, 2020. Following the previous two injections of Camrelizumab, the patient did not experience any discomfort and was discharged from the hospital. After two months of therapy, MRI was performed in the reexamination on February 16, 2021, revealing that the nodular enhancement slightly decreased in size. Good results were obtained in TACE combination with targeted agents and immunotherapy.

**Figure 3:**
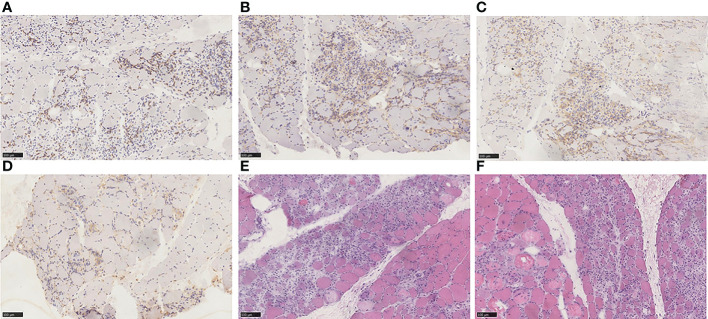
**(A)** Immunohistochemical staining for CD3. **(B)** Immunohistochemical staining for CD4. **(C)** Immunohistochemical staining for CD8. **(D)** Immunohistochemical staining for CD68. **(E)** HE staining. **(F)** HE staining. Endomysium expressed an abundance of CD68-positive cells and was also positive for CD3, CD4, and CD8 expression. The main pathological characteristics of skeletal muscle included necrosis of myofibers, regeneration, and inflammatory cell infiltration. Diagnosis: PD-1 mediated immune myositis with the medical history.

**Figure 4:**
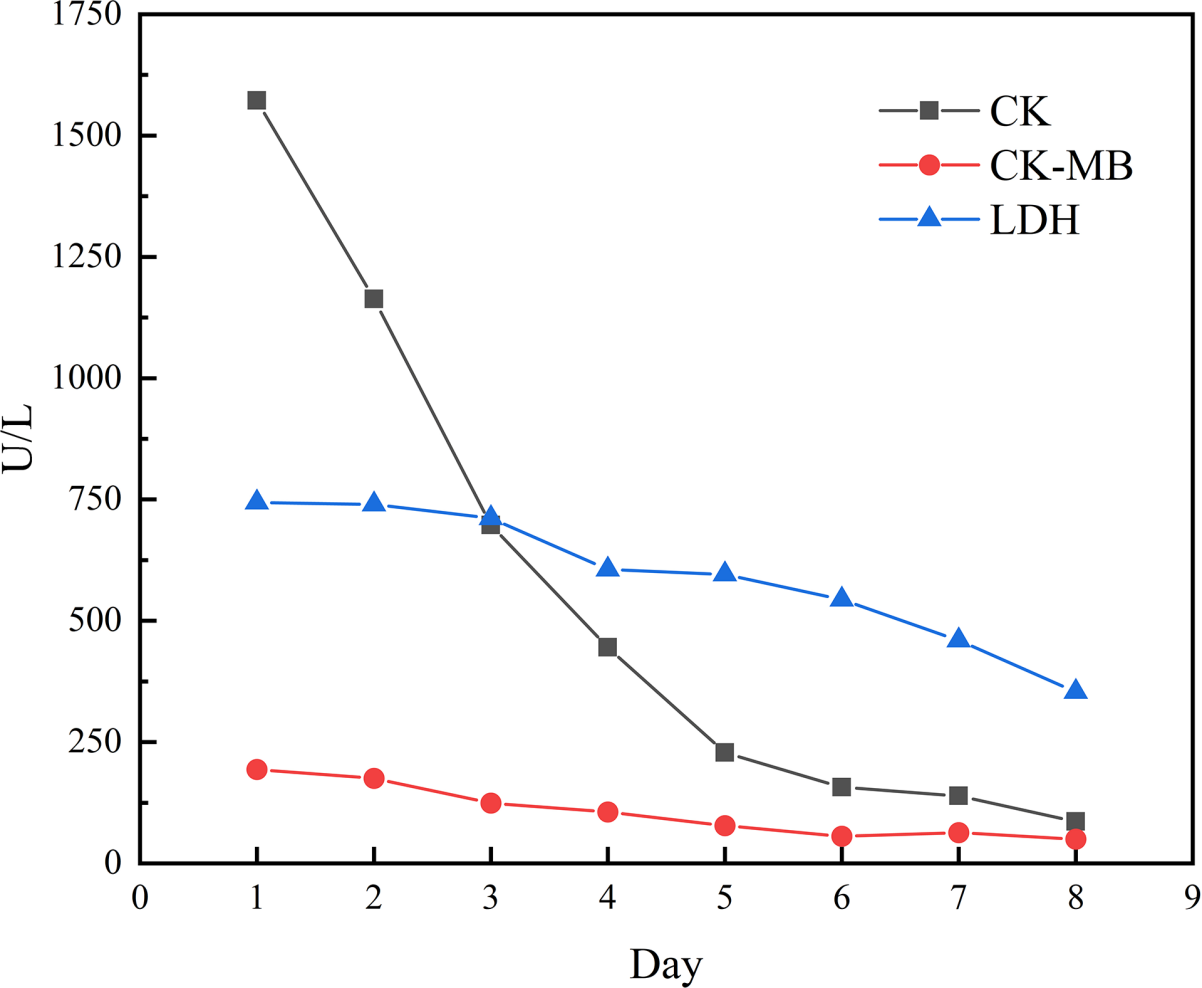
Changes of some indicators (2021/03/03-2021/03/10).

However, the patient had decreased muscle strength in both lower extremities with chest tightness, dyspnea, and expectoration (whitish sputum) on Feb 09, 2021. His performance status (PS) according to the Eastern Cooperative Oncology Group (ECOG) score was 2. Physical examination revealed left eyelid ptosis and grade IV muscle strength in both lower limbs. His cardiac color Doppler ultrasound and electrocardiography showed no obvious abnormalities. Biochemical parameters showed elevated cardiac biomarkers, creatine kinase (CK)1572.1 U/L (normal value 50-310 U/L), creatine kinase isoenzyme (CK-MB) 193.6 U/L (normal value 0-24.0 U/L), lactate dehydrogenase (LDH) 744.0 U/L (normal value 120-250 U/L), myoglobin >900 µg/L (normal value 23-11 µg/L), cardiac troponin (cTnI) level and brain natriuretic peptide (BNP) is normal. The following results evidenced abnormal liver function: alanine aminotransferase (ALT) 108.6 U/L (normal value 9-50 U/L) and AST 146.3 U/L (normal value 15-40 U/L). A muscle biopsy was taken from the left anterior tibialis muscle, and surgical findings showed inflammatory muscle (**Figure 3**). Based on his disease courses, clinical presentations, laboratory test results, and muscle biopsy findings, the diagnosis was ICIs injection-induced immune myocarditis and myositis accompanied by elevated transaminases, which is considered a grade 3/4 ICIs-induced adverse reaction. The patient discontinued immunotherapy and received a proper treatment immediately, including methylprednisolone sodium succinate intravenously for 7 days, with gradually decreasing doses. Polyene phosphatidylcholine and omeprazole sodium were also administered *via* an intravenous drip for supportive treatment. After 7 days of therapy, the patient stated that dyspnea and chest tightness significantly improved than before. His blood biochemical parameters, such as CK, CK-MB, and LDH have also gradually decreased (**Figure 4**). On Mar 12, 2021, re-examination *via* bio-chemistry indicated the following results: creatine kinase (CK) 82.6 U/L (normal value 50-310 U/L), creatine kinase isoenzyme (CK-MB) 50.0 U/L (normal value 0-24.0 U/L), lactate dehydrogenase (LDH) 353.8 U/L (normal value 120-250 U/L). The patient’s ECOG PS increasingly improved to 0, and no more episodes of the above symptoms. Because patient’s symptoms had improved significantly and the myocardial injury markers showed a significant decline. The patient was discharged from the hospital on day 9 and given oral methylprednisolone tablets (40mg daily, gradually decreasing doses) and proton pump inhibitor PPI.

One week after hospital discharge, reexamination showed that the patient’s CK-MB and LDH were all within the normal range. The patient had no obvious discomfort in the subsequent follow-up. At a follow-up examination one year later, magnetic resonance imaging (MRI) showed a subcapsular focus of the left lobe of the liver. The 1.7cm sized nodules on the hepatobiliary phase of MRI were performed with hypointense. Subsequently, the patient was treated by percutaneous microwave ablation. The stage of the operation was uneventful without complications. Rechallenges of targeted drugs or immunotherapy were not implemented in the subsequent therapy. To date, the patient generally fair well, and his liver have no new lesions.

## Discussion

Unlike other organs, the liver is an important component of the immune system that has a dual blood supply from the portal vein and the hepatic artery. It links the portal vasculature to the entire circulatory system. Local and systemic immune responses were associated with liver immune cells, such as hepatic stellate cells (HSCs), kupffer cells (KCs), dendritic cells (DC), myeloid cells, lymphocytes, and others (12). When varieties of antigens pass through, the liver can avoid systemic immune response and induce immune tolerance because of tight regulation derived from numbers of immunosuppressive cells, cytokines, and ligands (13). Liver tumor cells more easily escape immune surveillance due to the immune tolerance mechanism. On the other hand, the carcinogenesis of HCC is a multi-factor process resulting from the excess accumulation of aflatoxin and chronic infection of the hepatitis virus. Chronic hepatitis and liver fibrosis provide an immunosuppressive environment that benefits hepatocytes transforming and cancer cells reproducing. These mechanisms established the theoretical foundation for the development and clinical immunotherapy of HCC. Clinical studies with checkpoint-blocking antibodies targeting PD-1/PD-L1 and CTLA-4 are currently initiating. For advanced HCC patients, ICIs exhibit therapeutic benefits by enhancing T cells–mediated immunity. It is important to note that uncontrolled activation of cytotoxic T-cells has brought about many side effects in clinical immunotherapy. Because ICIs activate immunity throughout the body instead of just in the tumor, irAEs involve almost all organ systems. IrAEs of antibodies targeting PD-1 and/or PD-L1 include hypothyroidism and interstitial pneumonia, whereas irAEs of antibodies targeting CTLA-4 encompass mainly colitis and hypophysitis (14–16). However, most HCC patients have liver cirrhosis and portal hypertension. Their symptoms also overlap with the toxicity of ICIs. Likewise, the results of irAEs can increase liver and/or extrahepatic organ manifestations caused by cirrhosis.

Findings suggest that the odds of immune myocarditis range from 0.04% to 1.14%, but 25-50% lethal is high (17). Emma Matzen et al. (18). systematically reviewed the literature on 87 ICI-induced myocarditis patients in PubMed. They found that most cases were melanoma (n = 39), lung cancer (n = 19), renal cell cancer (n = 10), and thymoma cancer patients (n = 4). The median number of cycles of manifestation of cardiotoxic symptoms was 2 (range 1 to 13 cycles). The mechanisms underlying the association between ICIs treatment and myocarditis are still unclear. It suggested that ICIs treatment causes immune activation in HCC patients, concomitant with reducing T cell tolerance to normal tissues. The PD-1 knockout mice have an increased incidence of autoimmune dilated cardiomyopathy and mortality rates, with the deposition of diffuse antibodies (immunoglobulin G) in cardiomyocytes (19). PD-1 pathway plays an essential role in the regulation of the immune response of cardiac (19). In necropsy and histological analysis from ICIs-related fatal myocarditis patients, Cytotoxic T lymphocyte (CTL) infiltration and PD-L1 Inhibition are present in cardiomyocytes (9, 20). PD-L1 protein can be found in normal human cells. When they are integrated with the CTL receptors, the ability to protect overactivated cells can be activated (17, 20). Similar to what has been observed in humans, the infiltrated lymphocytes in monkey hearts include mainly CD4^+^ and CD8^+^ T cells, and immunohistochemical stains for PD-1 and PD-L1 are positive (21). Clinical signs and symptoms of immune myocarditis include non-specific ones of fever, shortness of breath, and fatigue. Typical symptoms include palpitations, chest pain, arrhythmias, heart failure, and even cardiogenic shock. Clinical signs and symptoms are not enough for a diagnosis of immune myocarditis. The clinical diagnosis should be concluded based on medication history, clinical manifestations, and laboratory results (cardiac injury marker, e. g.) rather than only based on clinical signs and symptoms. Troponin levels have been reported to be elevated in 94% of ICI-related myocarditis patients, and BNP/NT-proBNP levels can be raised in 66% of cases (22, 23). Whether troponin, BNP, or NT-proBNP, these biological indicators are not specific to the diagnosis of immune myocarditis. Echocardiography, cardiac MRI, or even cardiac biopsy may be necessary for some situations. We should also clearly diagnose any other cardiac diseases presented with the above symptoms. ICIs therapy should be immediately discontinued if patients are diagnosed with immune myocarditis. Depending on the severity of irAEs, patients should rapidly administer corticosteroids (1 to 2mg/kg) (24). Infliximab/anti-thymocyte globulin (ATG) is the attemptable treatment when high-dose corticosteroid therapy fails (24).

Jeffrey Aldrich et al. systematically reviewed the 9,088 cases receiving ICIs at the University of the Texas MD Anderson Cancer Center (10). The results demonstrated that the probability of immune myositis was 0.04% (36 cases). Other clinical symptoms, such as myocardial injury, myasthenia gravis, and respiratory failure, have been associated with half of the patient. The prognosis of single myositis patients is better than multiple symptoms patients. The mechanism of immune myositis may be similar to that of immune myocarditis. Because of shared antigens among myocardium and skeletal muscle, the loss of immunological tolerance induced by ICIs, and afterward caused irAEs. We performed a pathologic examination of the left anterior tibialis muscle biopsy samples. There were necrosis and regeneration of muscle fibers, and inflammatory cell infiltration was visible inside the muscle fibers. Mehdi Touat et al. (25) investigated the pathological results of 9 patients with immune myositis. Necrotic myofibers of varying degrees were noted in all patients; large amounts of macrophages were located in perimysial and endomysial tissue; CD4^+^, CD8^+^, and CD68^+^cells were infiltrated in endomysial tissue. Our pathological outcomes are in accordance with this finding (**Figure 3**). Patients with immune myositis could develop muscle aches and exhaustion as the initial symptom. However, it should be noted that the history of other neurologic disorders plays a crucial role in clinical work. Understanding muscle strength by physical examination and assessing the extent of muscle inflammation by laboratory tests also provide evidence for diagnosis. CK elevation occurs in almost all cases (26). T cells are stimulated by immune checkpoint suppression and manifest an aggressive response that results in irAEs (9). Skeletal muscle pathology of immune myositis included the infiltration of mononuclear cells, especially CD8^+^ T cells CD4^+^ T cells and B cells (27). These results in CD4^+^ T cell-mediated B cell activation and synthesis of pathogenic high-affinity autoantibodies (IgG1 and 3 or IgG4 subclass) (28). These antibodies were strongly associated with postsynaptic membrane clustering and structure maintenance of neuromuscular synapses by binding to the nicotinic acetylcholine receptor (AchR) or muscle-specific tyrosine-kinase (MuSK), etc (28). Ultimately, the whole process cause muscle strength decreased. Most cases of overlapping MG were positive in antibodies against acetylcholine receptors (AChR), but a small number of AChR-negative patients have symptoms of drooping eyelids and double vision (29, 30). Clinical unclear diagnosis of immune myositis should be confirmed with EMG or muscular biopsy. ICIs should be withdrawn once immune myositis is diagnosed. According to the severity of Adverse effects, patients should be treated with oral or intravenous corticosteroids. We could further use nonsteroidal anti-inflammatory drugs (NSAIDs) to treat muscle soreness after excluding some contraindications. If there is no appreciable benefit after 4-6 weeks, additional immunosuppression should also be taken into consideration (24).

Immune myositis and immune myocarditis could either occur alone or together. Clinicians should be vigilant of signs of other irAEs when one irAE is detected early. We summarized the characteristics of irAEs of HCC patients from case reports on PubMed (**Table 1**). We observed that the time from initial treatment with ICIs to irAEs was not long. Constantin et al. reported a case of Immune terminal ileitis due to an increasing dose of Nivolumab in a 58-year-old HCC man (33). Many patients were treated with targeted agents during immunotherapy (**Table 1**). This patient took antiangiogenic drugs (sorafenib) because of positive MVI and disease progression. Unlike ICIs, adverse targeted drug reaction was mostly found to be associated with the inhibition of vascular endothelial cell. The half-life of ICIs was 21 days, and antiangiogenic drugs’ half-life was 7 to 45h. The adverse targeted drug reaction occurs relatively early (1–2 weeks), while irAEs occur relatively late (within 3 months). The combination of Tyrosine kinase inhibitors (TKi) and ICIs expand the incidence of irAEs compared to ICIs monotherapy (41). Therefore, we discontinued sorafenib and Camrelizumab when our patient presented severe irAEs. The irAEs of liver cancer do not exhibit evident specificity compared to other tumor types. Offending drugs timely and steroid pulse therapy is an effective strategy for the treatment of most HCC patients. This patient was taken under an intravenous high-dose methylprednisolone sodium succinate therapy first. His CK-MB and LDH are still out of range after 7 days of intravenous steroid treatment. This suggests that mild inflammation and necrosis could exist in cardiac and skeletal muscles. But all myocardial injury markers decreased more than before. So we used a lower dose of methylprednisolone tablets and administered it orally. Treating with steroids until the cardiac function returns to baseline, then dose tape at least 4 weeks (42). Most irAEs were mild to moderate in severity and were reversible, but severe irAEs were still life-threatening. When the symptoms are relieved, the rechallenge of ICIs can be taken into account, but the decision to rechallenge must be interpreted with caution. Replacement of ICI is one of the optional schemes for immunosuppressant maintenance therapy (**Table 1**). When irAEs reach grade 3/4, rechallenge of ICIs is not generally recommended. Although rechallenge of ICIs after irAEs showed similar efficacy outcomes compared with initial ICI treatment (43). The reoccurrence odds of irAEs could be enhanced, and the reoccurrence time of irAEs could be advanced. For such cases, a closer long-term follow-up seems crucial. Patients could have permanent discontinuation once the irAEs have recurred (42).

**Table 1:** Review of HCC case reports published on PubMed.

Ref.	Sex	Age	ICIs	irAEs	Time from initial treatment with ICIs to irAEs	Treatment	Targeted agents	Rechallenge of ICIs	Subsequent therapy
(31)	Man	67	Nivolumab	Immune hepatitis	6 mo	Prednisone and MMF	No	NA	NA
(32)	Man	56	Sintilimab	Immune diabetes	24 wk	Insulin	No	Yes	Sintilimab
(33)	Man	58	Nivolumab	Immune terminal ileitis	18 mo (4 days after dose increased)	Methylprednisolone	Sorafenib	Yes	Nivolumab
(34)	Man	70	Nivolumab	Hypersensitivity reaction	14 d	Hydrocortisone and diphenhydramine	Sorafenib	Yes	Pembrolizumab
(35)	Female	70	Atezolizumab	Immune myocarditis	4 d	Methylprednisolone	Bevacizumab	NA	NA
(36)	Female	70	Atezolizumab	Encephalitis	10 d	Methylprednisolone and plasmapheresis	Bevacizumab	No	No, the patient died 76 d after initial treatment with ICIs due to multiorgan failure
(37)	Man	69	Nivolumab	Late-onset Stevens-Johnson syndrome	16 wk	Prednisone	No	NA	NA
(38)	Man	78	Sintilimab	Immune myocarditis	21 d	Methylprednisolone and gammaglobulin	Lenvatinib	NA	NA
(39)	Man	85	Atezolizumab	Diffuse alveolar hemorrhage (DAH)	5 cycles	Methylprednisolone (Steroid pulse therapy)	Bevacizumab	NA	NA
(40)	Man	32	Pembrolizumab	Stevens-Johnson syndrome and porokeratosis	3 cycles	Methylprednisolone and adalimumab	Sorafenib and apatinib	No	No, the patient died 1 week after discharge due to the progression of metastatic disease to the lungs

NA, Not available.

Before starting ICIs treatment, a comprehensive assessment is needed for judging the possibility of developing irAEs. This includes the patient’s general condition, the previous history of immune disease, laboratory tests and radiographic examinations. HCC patients were usually accompanied by liver cirrhosis or fatty liver which can cause abnormal liver function (44). Therefore, the etiology should be investigated promptly once abnormal liver function has occurred. Whether acute hepatitis or compression by tumors, active treatment is required before immunotherapy (45). Abnormal liver function may also cause endocrine/metabolic disorders (46). It is mandatory to evaluate thyroid function periodically. Chemotherapy is one of the most important treatment modalities for advanced HCC. But the combination of ICIs and chemotherapy can also expand the incidence of acute kidney injury (47, 48). Regular checkups, such as renal function tests or urine routines, need to be performed. Cutaneous adverse reactions can be detected by frequent and detailed physical examination. Further examination is needed once the diagnosis is suspected, such as enzyme-linked immunosorbent assays and immunofluorescence tests (49). In conclusion, early detection and early treatment are extremely important in the treatment of irAEs. Any new symptoms after ICI initiation could be irAEs. Presently, various therapeutic approaches combined with ICI therapy have become an essential treatment for HCC, and immunotherapy should not be abandoned easily because of potential irAEs. Adequate judgment, close monitoring, and early detection are needed clinically. Only in this way can we obtain the ideal clinical outcome for each individual.

## Data availability statement

The original contributions presented in the study are included in the article/supplementary material. Further inquiries can be directed to the corresponding author.

## Ethics statement

Written informed consent was obtained from the individual(s) for the publication of any potentially identifiable images or data included in this article. The participants provided their written informed consent to participate in this study.

## Author contributions

Conceptualization: HM and RW. Data curation: YX, WL, and JW. Writing-original draft: HM and WW. Writing-review & editing: KF and RW. All authors contributed to the article and approved the submitted version.
